# Mechanism of actions of Oncocin, a proline-rich antimicrobial peptide, in early elongation revealed by single-molecule FRET

**DOI:** 10.1007/s13238-017-0495-2

**Published:** 2017-12-18

**Authors:** Sijia Peng, Mengyi Yang, Rui Ning Sun, Yang Liu, Wenjuan Wang, Qiaoran Xi, Haipeng Gong, Chunlai Chen

**Affiliations:** 1 School of Life Sciences, Tsinghua University, Beijing, 100084 China; 2 Tsinghua-Peking Joint Center for Life Sciences, Tsinghua University, Beijing, 100084 China; 3 Beijing Advanced Innovation Center for Structural Biology, Tsinghua University, Beijing, 100084 China; 4 MOE Key Laboratory of Bioinformatics, Tsinghua University, Beijing, 100084 China; 5 Technology Center for Protein Sciences, Tsinghua University, Beijing, 100084 China

**Dear Editor,**

Proline-rich antimicrobial peptides (PrAMPs) are a class of antimicrobial peptides containing a high content of proline residues. PrAMPs selectively target Gram-negative bacteria through special transporters such as SmbA to enter cytoplasm (Mattiuzzo et al., 2007). On the other hand, PrAMPs present a low toxicity to mammalian cells, because they cannot effectively penetrate the mammalian cellular membrane (Hansen et al., 2012) or they are internalized through an endocytotic process to minimize interaction with cytosolic ribosomes (Tomasinsig et al., 2006). Therefore, PrAMPs are promising candidates to treat infections and to deliver drugs (Schmidt et al., 2016).

Oncocin represents a class of PrAMPs analogues modified from the *Oncopeltus* antibacterial peptides originally isolated from *Oncopeltus fasciatus* (milkweed bug). Oncocin has high antibacterial activity as its minimal inhibitory concentration (MIC) is around 1 μg/mL (Knappe et al., 2010). Hoffmann group discovered that Onc112 can strongly bind to the ribosome with nanomolar dissociation constants (Knappe et al., 2011). Structures of Onc112 bound ribosomes illustrated that Onc112 occupies three adjacent functional sites, including the upper ribosomal exit tunnel, peptidyl transferase center (PTC), and ribosomal aminoacyl-tRNA (aa-tRNA) binding site (A-site) (Roy et al., 2015; Seefeldt et al., 2015), which suggests that Onc112 could hinder entrance of aa-tRNA into the ribosome. Structures of Onc112 bound ribosomes provide foundation to thoroughly elucidate its antibacterial mechanisms. Here, we apply single-molecule fluorescence resonance energy transfer (smFRET) assays we have developed (Peng et al., 2017) to characterize how Onc112 affects translation in several early elongation cycles.

Firstly, we characterized how Onc112 affects percentage of active ribosomes and elongation rates via smFRET between two adjacent tRNAs. Six different messenger RNAs (mRNAs) were used (Table S1). They all encode a common VF motif, which was placed at the 0, 2, 4, 6, 8, or 10 codon positions after the AUG start codon. Each active ribosome translating through the common VF motif produced one smFRET event as shown in Fig. 1A and 1B. The number of Cy5-V/Cy3-F FRET events captured in the presence of Onc112, normalized by the number of FRET events in the absence of Onc112, was used to define the percentage of active ribosomes translating through the Phe codon (Fig. 1C). We found that, with Onc112, percentage of active ribosomes gradually decreases from ~70% in the second elongation cycle to ~30% in the eighth cycle. The Cy5-only state, shown between arrows 1 and 2 in Fig. 1A, corresponds to a full elongation cycle. Its dwell times were almost doubled in the presence of Onc112 (Figs. 1B, 1D, and S1), which clearly demonstrated that Onc112 slows overall elongation rate. In addition, Onc112 significantly hindered elongation at 1 μmol/L or higher concentration (Fig. S2), which agrees with that its MIC is around 2 μmol/L against *E*. *coli* (Knappe et al., 2011). In all, Onc112 slows overall elongation rates and reduces percentage of active ribosomes in early elongation cycles.

**Figure 1:**
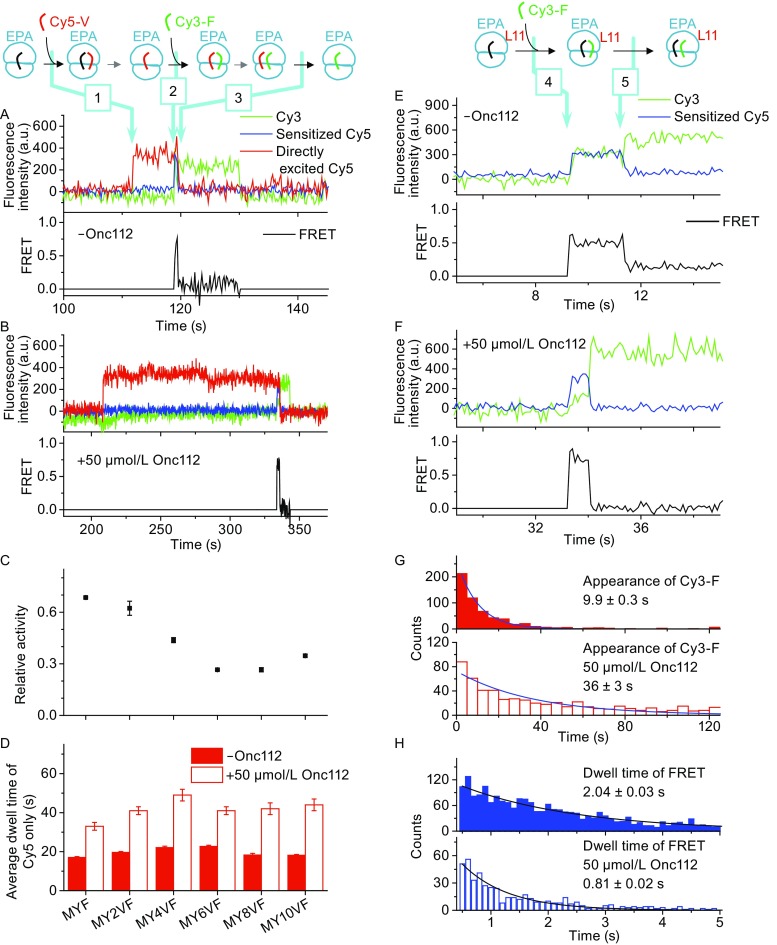
**smFRET reveals that Onc112 inhibits elongation**. (A and B) Typical real-time elongation traces measured using Cy5-V and Cy3-F without (A) or with (B) 50 μmol/L Onc112, for ribosomes programmed with mRNAs containing a common VF motif (Table S1). Cy5-V, Cy3-F, and other necessary components were flowed onto microscope slides at time *t* = 0. In addition to Cy3 fluorescence (green) and sensitized emission of Cy5 (FRET, blue) under 532 nm laser excitation, Cy5 fluorescence (red) was collected under alternating 640 nm laser excitation. No fluorescence signal was detected until accommodation of Cy5-V (arrow 1). The Cy5-only state lasted for a full elongation cycle until accommodation of Cy3-F, the next aa-tRNA (arrow 2). When both Cy5-V and Cy3-F were present on the ribosome, fluorescence intensities of Cy3, sensitized Cy5 due to FRET from Cy3, and Cy5 directly excited by 640 nm laser were all detected (between arrows 2 and 3). Cy5 and FRET signals disappeared when Cy5-V dissociated from the E-site (arrow 3). (C) Relative activity of ribosomes elongated to the VF motif, defined by the number of Cy5-V/Cy3-F FRET events captured in the presence of Onc112 normalized by the number of FRET events captured in the absence of Onc112. (D) Average dwell times of the Cy5-only state in the absence and presence of Onc112 for six different mRNAs, which were calculated by exponential fitting of dwell time distributions shown in Fig. S1. (E and F) Typical real-time elongation traces of ribosomes programmed with mRNA MVF measured using Cy5-L11 and Cy3-F without (E) or with (F) 50 μmol/L Onc112. Cy3-F and other necessary components were flowed onto microscope slides at time *t* = 0. Only Cy3 fluorescence (green) and sensitized emission of Cy5 (FRET, blue) were collected under 532 nm laser excitation. Formation of PRE-translocation complexes after Cy3-F accommodation led to appearance of Cy3 signal and high FRET efficiency between Cy5-L11 and Cy3-F (arrow 4). Translocation of Cy3-F from the A-site to P-site increased distance between Cy5-L11 and Cy3-F which caused decrease of FRET (arrow 5). (G and H) Dwell time distributions of POST-translocation (G, measured from *t* = 0 to arrow 4) and PRE-translocation complexes (H, measured between arrows 4 and 5) without Onc112 (solid bars) or with Onc112 (hollow bars). Time constants calculated by exponential fitting of dwell time distributions were listed with S.E. of fitting results. Number of events included are 599 (top) and 635 (bottom) in (G) and 2,536 (top) and 552 (bottom) in (H)

We examined how deletions in Onc112 affect its ability to inhibit elongation (Fig. S3). OncΔNr, a C-terminally truncated derivative, affected elongation in a similar manner as Onc112. However, OncΔVD, an N-terminally truncated derivative, caused minor inhibition. Our results agreed with previous findings that the N-terminus of Onc112 plays a key role in binding and deactivating the ribosome and its C-terminus mainly functions in cellular uptake (Seefeldt et al., 2015). Three control peptides, which were made of randomized or truncated Onc112 sequence, showed almost no inhibition on elongation (Fig. S4).

Next, we examined how rates of aa-tRNA delivery and translocation are effected by Onc112 via smFRET between ribosomal protein L11 and aa-tRNA. We mainly focused on the second elongation cycle of ribosomes programmed with mRNA MVF. Consistent with our previous findings, a high L11-tRNA FRET state (energy transfer efficiency *E* = 0.4–0.8) appeared when Cy3-F was delivered into the A-site (arrow 4 in Fig. 1E). After several seconds, L11-tRNA FRET decreased significantly (*E* = 0.1–0.2) when Cy3-F was translocated to the P-site (arrow 5 in Fig. 1E). The rate of aa-tRNA delivery in the second elongation cycle of ribosomes programmed with MVF, calculated from the delay time between injection of Cy3-F (defined as *t* = 0) and the appearance of steady Cy3 and FRET intensities (arrow 4 in Fig. 1E), markedly decreased from 12.6 ± 0.4 s^−1^(μmol/L)^−1^ to 3.5 ± 0.3 s^−1^(μmol/L)^−1^ in the presence of Onc112 (Fig. 1G). Onc112 also decreased delivery rates of aa-tRNA in the first elongation cycle (Fig. S5). Our findings agreed with previous speculation derived from structural studies, which proposed that Onc112 could hinder entrance of aa-tRNA into the ribosome (Roy et al., 2015; Seefeldt et al., 2015). On the other hand, translocation rate in the second elongation cycle of ribosomes programmed with MVF, calculated from dwell time of high L11-tRNA FRET state (Fig. 1E between arrows 4 and 5), increased from 0.49 ± 0.01 s^−1^ to 1.23 ± 0.03 s^−1^ in the presence of Onc112 (Fig. 1F and 1H). We found that conformational dynamics of ribosomal PRE-translocation (PRE) complexes prior to translocation were also affected by Onc112. 50 μmol/L Onc112 destabilized the classical PRE state by decreasing its dwell time from 0.19 ± 0.01 s to 0.14 ± 0.01 s and stabilized the hybrid PRE states by increasing its dwell time from 0.20 ± 0.01 s to 0.26 ± 0.02 s, which would facilitate the following translocation step as shown by previous smFRET studies. The apparent rates of aa-tRNA delivery and translocation captured here, in the absence of Onc112, were close to our previous reported values and fit well within the range of 3.5–30 s^−1^(μmol/L)^−1^ (Pape et al., 1998; Johansson et al., 2008) and 0.4–2 s^−1^ (Pan et al., 2007), respectively, reported by other groups. In summary, we concluded that Onc112 mainly impairs delivery of aa-tRNA to the ribosome.

Aa-tRNAs are delivered to and selected by the ribosome via a multi-step process (Rodnina and Wintermeyer, 2001), which can be dissected using smFRET between P-site tRNA and incoming aa-tRNA at high data collecting rate (Geggier et al., 2010). We used a similar method to examine how Onc112 impairs delivery of aa-tRNA in details (Fig. 2A and 2B). Duration of FRET events clearly exhibited two components whose lifetimes were about ten-fold difference from each other (Fig. 2C and Table S2). In the absence of Onc112, the majority of FRET traces belonged to the long-lived component, which evolved from a transient low FRET state (*E* = 0.2) to a steady high FRET state (*E* = 0.5–0.6) until fluorescence signals disappeared due to photo-bleaching (Fig. 2A and 2C–F top panel). Onc112 significantly increased the proportion of the short-lived component, which evolved from a transient medium FRET state (*E* = 0.4) to another transient high FRET state (*E* = 0.6) (Fig. 2B and 2C–F middle panel). For these short-lived events, disappearance of FRET was caused by dissociation of Cy3-F from the ribosome because its disappearance rate was an order of magnitude faster than photo-bleaching rate. When we performed the same FRET measurements using ribosomes programmed with mRNA MVL (Table S1) which contained a CUC tri-nucleotide sequence, a near-cognate codon for Phe-tRNA^Phe^, in the A-site, the majority of FRET traces belonged to the short-lived component (Fig. 2C–F bottom panel). At the same experimental conditions and instrumental configuration, no FRET event was captured when ribosomes contained non-cognate codons in the A-site. Together, these results indicated that the short-lived FRET events captured here represent aa-tRNA rejection events, which are likely to happen during the proofreading step of tRNA selection (Rodnina and Wintermeyer, 2001; Geggier et al., 2010). Onc112 significantly hinders aa-tRNA delivery by increasing rejection rate of the correct cognate aa-tRNA. About half of cognate aa-tRNA binding events are mistakenly rejected by the ribosome, which leads to slower overall aa-tRNA delivery rate in the presence of Onc112. Using mRNA MY6VF, we found similar behaviors that Onc112 increased rejection rate of cognate aa-tRNA at the eighth elongation cycle (Table S3).

**Figure 2:**
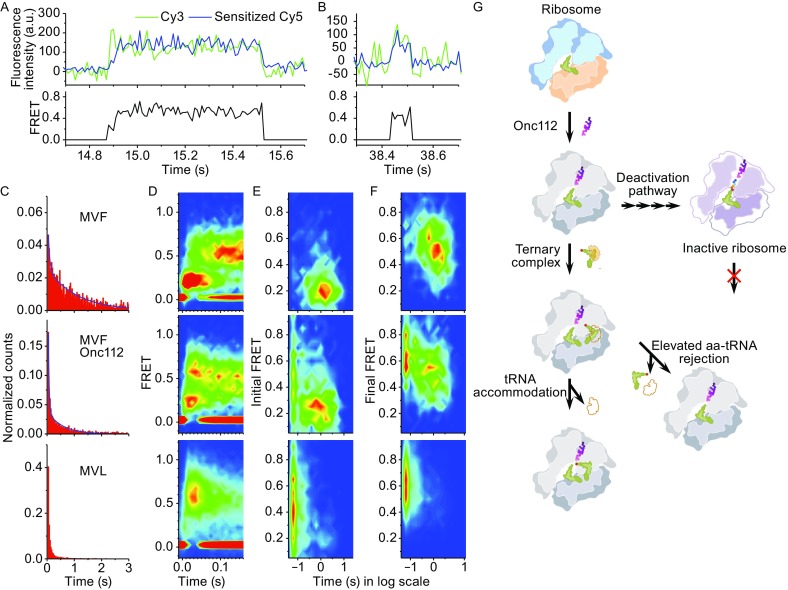
**Onc112 inhibits aa-tRNA delivery and its proposed mechanisms**. (A and B) Two types of typical real-time traces recorded while injecting 8 nmol/L Cy3-F ternary complex into flow channel, defined as time *t* = 0, containing immobilized ribosomal POST-translocation complexes with Cy5-V in the P-site. Both long (A) and short (B) FRET events were captured using ribosomes programmed with mRNAs MVF or MVL, whose A-sites contain cognate or near-cognate codons for Cy3-F, respectively. Only Cy3 fluorescence (green) and sensitized emission of Cy5 (FRET, blue) were collected under 532 nm laser excitation. (C) Dwell time distributions of all FRET events captured using mRNA MVF without (top panel) or with (middle panel) Onc112, or mRNA MVL without Onc112 (bottom panel). Details of fitting results were listed in Table S2. The same layout applies in (D–F). (D) FRET probability density plots as a function of time. All traces are aligned to the beginning of FRET events as *t* = 0, when both Cy3 and FRET signals appear simultaneously. (E and F) Heat maps constructed from initial FRET value when FRET events appear (E) or final FRET value right before FRET events disappear (F) vs. dwell times of FRET events. Each single-molecule event contributed as a single point in two-dimensional plots of FRET values *vs.* time. Heat maps were two-dimensional histograms made from all data points. Please notice that the top and bottom panels both contain one dominate population, whereas the middle panel has two populations. Number of events used here were listed in Table S2. (G) Proposed scheme to elucidate mechanisms of Onc112 in early elongation cycles. Two aspects have been highlighted. 1) Onc112 increases cognate aa-tRNA rejection rates, which leads to slower elongation rates. 2) Onc112 gradually deactivates 70% of ribosomes within 7–8 elongation cycles

When nascent peptide chain is elongated, Onc112 would have to move towards the exit of the ribosomal polypeptide exit tunnel to avoid physically clash with nascent peptide chain (Roy et al., 2015; Seefeldt et al., 2015). We performed a molecular dynamics (MD) simulation to estimate non-bonded interaction energy between Onc112 and the polypeptide exit tunnel when it moves towards the exit (Fig. S6). Our simulation indicated that previous structures (Roy et al., 2015; Seefeldt et al., 2015) captured the most stable binding site of Onc112, which gives the lowest interaction energy in our MD simulation. When Onc112 moves towards the exit site from its most stable site, interaction energy gradually increases until it reaches a plateau at ~15 Å displacement (6–7 residues). Therefore, Onc112 causes extra energy cost to extend nascent peptide in the first 6–7 elongation cycles, which correlates with the gradual decrease of the percentage of active ribosomes in the first 8 cycles captured by smFRET (Fig. 1C). Once the nascent peptide is longer than 8 residues (>19 Å in Fig. S6), there is no extra energy cost to further move Onc112 towards the exit and the percentage of active ribosomes remains almost constant (Fig. 1C).

Previous structural and biochemical studies suggested that Onc112 mainly acts in the elongation phase of protein synthesis (Roy et al., 2015; Seefeldt et al., 2015). Consistent with previous model, we found that Onc112 only moderately inhibits the formation of initiation complexes (Fig. S7). We proposed a reaction scheme to illustrate mechanisms of Onc112 in early elongation (Fig. 2G), in which Onc112 binds within the polypeptide exit tunnel and moves towards the exit when nascent peptide gets elongated. We have revealed two major aspects. Firstly, Onc112 greatly accelerates rejection rate of cognate tRNA during aa-tRNA delivery. The percentage of rejected cognate tRNA is similar to the percentage of successfully accommodated tRNA, which implies that rate of rejection is elevated about an order of magnitude and is approximately the same as the rate of aa-tRNA accommodation (Rodnina and Wintermeyer, 2001). Elevated rejection rate of cognate tRNA leads to slow overall elongation rate, which would significantly hinder growth and survival of cells. Secondly, Onc112 gradually deactivates translating ribosomes in the early elongation cycles. About ~70% of ribosomes are active in the second elongation cycle, which further decrease to ~30% in the eighth elongation cycle. Strong correlation between interaction energy landscape estimated from MD simulation (Fig. S6) and the percentage of active ribosomes measured from smFRET assays (Fig. 1C) supports our model, in which Onc112 serves as a peptide exit tunnel blocker. Interactions between Onc112 and the ribosome introduce extra energy cost in the first 6–7 elongation cycles, which reduce elongation rates and gradually deactivate ~70% of ribosomes. The model we proposed here could represent conserved mechanism of action used by many other PrAMPs (Gagnon et al., 2016), which also provides guide to design and optimize future antibiotic drugs.

## Electronic supplementary material

Below is the link to the electronic supplementary material.

Supplementary material 1 (PDF 733 kb)
