# Supplementary material for: Mechanism of actions of Oncocin, a proline-rich antimicrobial peptide, in early elongation revealed by single-molecule FRET
Source: Protein Cell. 2017 Dec 18;9(10):890–5. doi: 10.1007/s13238-017-0495-2 (PMC6160386; doi:10.1007/s13238-017-0495-2)
Supplement: Supplementary file 1 — Supplementary material 1 (PDF 733 kb) [file 13238_2017_495_MOESM1_ESM.pdf]

Supplementary Information for

Mechanism of actions of Oncocin, a proline-rich antimicrobial peptide, in early elongation revealed by single-molecule FRET

Sijia Peng [a,b,c], Mengyi Yang [a,b,c], Rui Ning Sun [a,c,d], Yang Liu [a], Wenjuan Wang [a,e], Qiaoran Xi [a], Haipeng Gong [a,c,d], Chunlai Chen [a,b,c,*]

a, School of Life Sciences; b, Tsinghua-Peking Joint Center for Life Sciences; c, Beijing Advanced Innovation Center for Structural Biology; d, MOE Key Laboratory of Bioinformatics; e, Technology Center for Protein Sciences, Tsinghua University, Beijing, China.

**Correspondence: chunlai@mail.tsinghua.edu.cn*

Methods and Materials

Ribosome preparation

Unlabeled 70S and 70S with Cy5-labeled L11 (70S-L11^{Cy5}) ribosomes were prepared according to published procedures (Subramanian and Dabbs, 1980; Rodnina and Wintermeyer, 1995; Chen et al., 2011; Stevens et al., 2012). Initiation complexes (ICs) were formed by mixture of 70S or 70S-L11^{Cy5} ribosomes with mRNA, initiation factors, and tRNA^{fMet} in TAM$_{15}$ buffer (15 mM MgAc$_2$, 50 mM Tris-HCl pH 7.5, 30 mM NH$_4$Cl, 70 mM KCl, and 1 mM dithiothreitol) at 37 ℃ for 25 min.

Charged and Labeled tRNA preparation

Amino acid specific tRNAs, *E. coli* tRNA^{fMet} (MP Biomedicals), *E. coli* tRNA^{Tyr} (Sigma), yeast tRNA^{Phe} (Sigma), *E. coli* tRNA^{Val} (Sigma) were prepared using the reduction, charging and labeling protocol as described (Pan et al., 2009; Kaur et al., 2011). Stoichiometries of fluorophore/tRNA labeling varied from 0.7 – 1.2 probe per tRNA (Kaur et al., 2011). Ternary complexes were formed by incubating 8 µM EF-Tu, 1 µM charged tRNAs, 3 mM GTP, 1.3 mM phosphoenolpyruvate, and 5 µg/mL pyruvate kinase in TAM$_{15}$ buffer for 15 min at 37 ℃.

Oncocins

Onc112, whose sequence is VDKPPYLPRPRPPRrIYNr-NH2 (r stands for D-arginine), and its derivatives were chemically synthesized by GL Biochem (Shanghai) Ltd and their purities (>95%) were verified by HPLC and mass spectrometry (Fig. S8). The other Onc112 derivatives and their sequences are: OncΔVD (KPPYLPRPRPPRrIYNr-NH2), OncΔNr (VDKPPYLPRPRPPRrI), Onc-N7 (VDKPPYL), Onc-C12 (PRPRPPRrIYNr), and Onc-rand (RNRYRIPLPYRKPDRVPPP). 50 µM of peptides were used in experiments unless otherwise noted.

mRNA preparation

mRNA MF, MVF, and MVL were purchased as 5’-biotinylated derivatives (Dharmacon RNAi Tech.) and used as received. mRNAs MY2VF, MY4VF, MY6VF, MY8VF, and MY10VF were prepared via in vitro transcription and 3’-biotinylation, as described before (Chen et al., 2013). Their sequences were listed in Table S1.

Acquisition and analysis of smFRET data

Initiation complexes (ICs) were specifically attached to microscope flow cells via biotinylated mRNA to PEG-passivated slides, decorated with biotin-PEG and streptavidin. Ribosomes were pre-equilibrated with Onc112 for 5 min before single-molecule recordings when needed. 5 min incubation time is sufficient for Onc112 to enter its binding sites on the ribosome and to inhibit elongation (Table S4, Fig. S5). smFRET experiments (Chen et al., 2011; Chen et al., 2013) were performed at 23 ℃ in TAM_{15} buffer (15 mM $MgAc_2$, 50 mM Tris-HCl pH 7.5, 30 mM NH_4Cl, 70 mM KCl, and 1 mM dithiothreitol) with an oxygen scavenging system, containing 3 mg/mL glucose, 100 μg/mL glucose oxidase (Sigma-Aldrich), 40 μg/mL catalase (Roche), 1 mM cyclooctatetraene (COT, Sigma-Aldrich), 1 mM 4-nitrobenzylalcohol (NBA, Sigma-Aldrich), 1.5 mM 6-hydroxy-2,5,7,8-tetramethyl-chromane-2-carboxylic acid (Trolox, Sigma-Aldrich – added from a concentrated DMSO stock solution). 50 μM of Onc112 was used in experiments unless otherwise noted.

Single-molecule spectroscopic microscopy was performed on a home-built objective-type TIRF microscope, whose configuration was described elsewhere in details (Peng et al., 2017). Collection of real-time translation traces began 10 s prior to injecting labeled and unlabeled ternary complexes, 4 μM EF-G, and 2 mM GTP into flow chambers containing immobilized ribosomes, and was carried out without further washing. To capture smFRET events at rate of 10 ms/frame, exposure area of EMCCD was truncated to 512 pixels by 190 pixels. All smFRET movies were collected using Cell Vision software (Beijing Coolight Technology).

Molecular dynamics (MD) simulation

The atomic structure of the *Thermus thermophilus* 70S ribosome was chosen from the Protein Date Bank (PDB code: 4Z8C) (Roy et al., 2015). Only a part of the 50S subunit surrounding the polypeptide exit tunnel was adopted for molecular dynamics (MD) simulation. The complex was solvated by 172449 water molecules in a rectangular solvent box with a distance of 8 Å around the complex. The system was electrostatically neutralized by 100 mM KCl. All simulations were performed by NAMD 2.10 (Phillips et al., 2005) in an NPT ensemble using CHARMM36 force field (MacKerell et al., 1998; MacKerell et al., 2004; Vanommeslaeghe et al., 2010; Best et al., 2012). The van der Waals interaction was smoothly switched to zero from 8 Å to 10 Å, and all interactions beyond 10 Å were ignored from energy calculation. Long-range electrostatics was calculated by particle mesh Ewald method (Essmann et al., 1995). MD simulations were controlled at 310 K using Langevin thermostat (Tu et al., 1995) and at 1 atm using Langevin piston(Martyna et al., 1994; Feller et al., 1995). The time step was chosen as 2 fs and the SETTLE algorithm(Miyamoto and Kollman, 1992) was used to enable the rigid bonds connected to all hydrogen atoms. The backbone atoms of both nucleic acid and protein that were located 10 Å

from the tunnel were harmonically restrained with a force constant of 5 kcal/mol/Å^2 in the whole simulation. After 1.5 ns of pre-equilibration, steered MD (SMD) simulation was applied in the system for 2 ns, to guide the movement of peptide. The forces were applied on the backbone of peptide with a force constant of 1 kcal/mol/Å^2 and a velocity of 0.00005 Å/fs, in the direction pointing from the weight center of four N-terminal residues to that of the rest residues. The snapshots from the SMD trajectory were relaxed by 1000 steps of energy minimization and then by 1 ns of equilibration with the peptide backbone restrained (using a force constant of 4 kcal/mol/Å^2). The SMD and equilibration trajectories were then used to calculate non-bonded interaction energy between peptide and the tunnel (include nearby proteins), against the traveling distance of the N-terminus of peptide (compared with crystal structure, which is defined as 0 displacement) (Fig. S6). With the rise of distance, the energy landscape showed a general trend to increase and reached a plateau around 15 Å.

References:

Best, R.B., Zhu, X., Shim, J., Lopes, P.E., Mittal, J., Feig, M., and Mackerell, A.D., Jr. (2012). Optimization of the additive CHARMM all-atom protein force field targeting improved sampling of the backbone phi, psi and side-chain chi(1) and chi(2) dihedral angles. *Journal of chemical theory and computation* **8,** 3257-3273. doi:10.1021/ct300400x

Chen, C.L., Stevens, B., Kaur, J., Cabral, D., Liu, H.Q., Wang, Y.H., Zhang, H.B., Rosenblum, G., Smilansky, Z., Goldman, Y.E., *et al.* (2011). Single-molecule fluorescence measurements of ribosomal translocation dynamics. *Mol Cell* **42,** 367-377.

Chen, C.L., Zhang, H.B., Broitman, S.L., Reiche, M., Farrell, I., Cooperman, B.S., and Goldman, Y.E. (2013). Dynamics of translation by single ribosomes through mRNA secondary structures. *Nature Structural & Molecular Biology* **20,** 582-+.

Essmann, U., Perera, L., Berkowitz, M.L., Darden, T., Lee, H., and Pedersen, L.G. (1995). A Smooth Particle Mesh Ewald Method. *J Chem Phys* **103,** 8577-8593. doi:Doi 10.1063/1.470117

Feller, S.E., Zhang, Y.H., Pastor, R.W., and Brooks, B.R. (1995). Constant-Pressure Molecular-Dynamics Simulation - the Langevin Piston Method. *J Chem Phys* **103,** 4613-4621. doi:Doi 10.1063/1.470648

Kaur, J., Raj, M., and Cooperman, B.S. (2011). Fluorescent labeling of tRNA dihydrouridine residues: Mechanism and distribution. *Rna* **17,** 1393-1400.

MacKerell, A.D., Bashford, D., Bellott, M., Dunbrack, R.L., Evanseck, J.D., Field, M.J., Fischer, S., Gao, J., Guo, H., Ha, S., *et al.* (1998). All-atom empirical potential for molecular modeling and dynamics studies of proteins. *The journal of physical chemistry. B* **102,** 3586-3616. doi:10.1021/jp973084f

MacKerell, A.D., Jr., Feig, M., and Brooks, C.L., 3rd (2004). Improved treatment of the protein backbone in empirical force fields. *Journal of the American Chemical Society* **126,** 698-699. doi:10.1021/ja036959e

Martyna, G.J., Tobias, D.J., and Klein, M.L. (1994). Constant-Pressure Molecular-Dynamics Algorithms. *J Chem Phys* **101,** 4177-4189. doi:Doi 10.1063/1.467468

Miyamoto, S., and Kollman, P.A. (1992). Settle - an Analytical Version of the Shake and Rattle

Algorithm for Rigid Water Models. *Journal of computational chemistry* **13,** 952-962. doi:Doi 10.1002/Jcc.540130805

Pan, D.L., Qin, H.O., and Cooperman, B.S. (2009). Synthesis and functional activity of tRNAs labeled with fluorescent hydrazides in the D-loop. *Rna* **15,** 346-354.

Peng, S., Sun, R., Wang, W., and Chen, C. (2017). Single-Molecule Photoactivation FRET: A General and Easy-To-Implement Approach To Break the Concentration Barrier. *Angewandte Chemie* **56,** 6882-6885. doi:10.1002/anie.201702731

Phillips, J.C., Braun, R., Wang, W., Gumbart, J., Tajkhorshid, E., Villa, E., Chipot, C., Skeel, R.D., Kale, L., and Schulten, K. (2005). Scalable molecular dynamics with NAMD. *Journal of computational chemistry* **26,** 1781-1802. doi:10.1002/jcc.20289

Rodnina, M.V., and Wintermeyer, W. (1995). GTP consumption of elongation-factor Tu during translation of heteropolymeric messenger-RNAs. *P Natl Acad Sci USA* **92,** 1945-1949.

Roy, R.N., Lomakin, I.B., Gagnon, M.G., and Steitz, T.A. (2015). The mechanism of inhibition of protein synthesis by the proline-rich peptide oncocin. *Nat Struct Mol Biol* **22,** 466-469. doi:10.1038/nsmb.3031

Stevens, B., Chen, C., Farrell, I., Zhang, H., Kaur, J., Broitman, S.L., Smilansky, Z., Cooperman, B.S., and Goldman, Y.E. (2012). FRET-based identification of mRNAs undergoing translation. *PLoS ONE* **7,** e38344.

Subramanian, A.R., and Dabbs, E.R. (1980). Functional-studies on ribosomes lacking protein L1 from mutant Escherichia-coli. *European Journal of Biochemistry* **112,** 425-430.

Tu, K., Tobias, D.J., and Klein, M.L. (1995). Constant pressure and temperature molecular dynamics simulation of a fully hydrated liquid crystal phase dipalmitoylphosphatidylcholine bilayer. *Biophysical journal* **69,** 2558-2562. doi:10.1016/S0006-3495(95)80126-8

Vanommeslaeghe, K., Hatcher, E., Acharya, C., Kundu, S., Zhong, S., Shim, J., Darian, E., Guvench, O., Lopes, P., Vorobyov, I., *et al.* (2010). CHARMM general force field: A force field for drug-like molecules compatible with the CHARMM all-atom additive biological force fields. *Journal of computational chemistry* **31,** 671-690. doi:10.1002/jcc.21367

Table S1. mRNA sequences

Name	Sequence
MF	GGG AAU UCA AAA AUU UAA AAG UUA AUA AGG AUA CAU ACU AUG UUC GUG CGU UAU GAA UAU GAA UAU
MVF	GGG AAU UCA AAA AUU UAA AAG UUA AUA AGG AUA CAU ACU AUG GUG UUC CGU UAU GAA UAU GAA UAU
MY2VF	GGG AAU UCA AAA AUU UAA AAG UUA AUA AGG AUA CAU ACU AUG UAU UAC GUG UUC CGU UAA CGC GUC UGC AGG CAU GCA AGC AAA AAA C
MY4VF	GGG AAU UCA AAA AUU UAA AAG UUA AUA AGG AUA CAU ACU AUG UAU UAC UAU UAC GUG UUC CGU UAA CGC GUC UGC AGG CAU GCA AGC AAA AAA C
MY6VF	GGG AAU UCA AAA AUU UAA AAG UUA AUA AGG AUA CAU ACU AUG UAU UAC UAU UAC UAU UAC GUG UUC CGU UAA CGC GUC UGC AGG CAU GCA AGC AAA AAA C
MY8VF	GGG AAU UCA AAA AUU UAA AAG UUA AUA AGG AUA CAU ACU AUG UAU UAC UAU UAC UAU UAC UAU UAC GUG UUC CGU UAA CGC GUC UGC AGG CAU GCA AGC AAA AAA C
MY10VF	GGG AAU UCA AAA AUU UAA AAG UUA AUA AGG AUA CAU ACU AUG UAU UAC UAU UAC UAU UAC UAU UAC UAU UAC GUG UUC CGU UAA CGC GUC UGC AGG CAU GCA AGC AAA AAA C
MVL	GGG AAU UCA AAA AUU UAA AAG UUA AUA AGG AUA CAU ACU AUG GUG CUC CGU UAU GAA UAU GAA UAU

Underlined sequences highlight regions from initiator codon until Phe or Leu codon.

Table S2. Dwell times of FRET events recorded at 10 ms per frame while delivering Cy3-F ternary complex without or with 50 μM Onc112 to pre-formed POST-translocation (POST) complex with Cy5-V in the P-site.

Experimental conditions	Number of FRET events	Lifetime and percentage of sub-populations (sec)	Weighted average lifetime (sec)
MVF	1422	0.052 ± 0.005 (9.4%)<br>1.07 ± 0.03 (90.6%)	0.97 ± 0.02
MVF + 50 μM Onc112	1689	0.0278 ± 0.0007 (51.3%)<br>0.55 ± 0.02 (48.7%)	0.282 ± 0.008
MVL	3294	0.0225 ± 0.0004 (79.8%)<br>0.147 ± 0.004 (20.2%)	0.048 ± 0.001

Table S3. Dwell times of FRET events recorded at 10 ms per frame while delivering Cy3-F ternary complex without or with Onc112 to pre-formed POST complex with mRNA MY6VF elongated to the 8th elongation cycle and Cy5-V in the P-site.

Experimental conditions	Number of FRET events	Lifetime and percentage of sub-populations (sec)	Weighted average lifetime (sec)
MY6VF	909	0.048 ± 0.007 (4.4%)<br>0.69 ± 0.02 (95.6%)	0.66 ± 0.02
MY6VF<br>+ 50 μM Onc112	1335	0.048 ± 0.002 (31.3%)<br>0.71 ± 0.02 (68.7%)	0.50 ± 0.02

Table S4. Relative active of initiation complexes (IC) to have aa-tRNA (Cy3-F) accommodated in the presence of 50 μM Onc112.

Experimental conditions [a]	Relative activity (%) [b]
without Onc112 during IC formation	73 ± 2
with 50 μM Onc112 during IC formation	69 ± 6

[a] In both conditions, 50 μM Onc112 was incubated with pre-formed IC for 5 min before adding Cy3-F.

[b] Activities were quantified by the number of Cy3 spots after incubating Cy3-F with immobilized IC for 3 minutes. Activity in the absence of Onc112 was normalized to 100%.

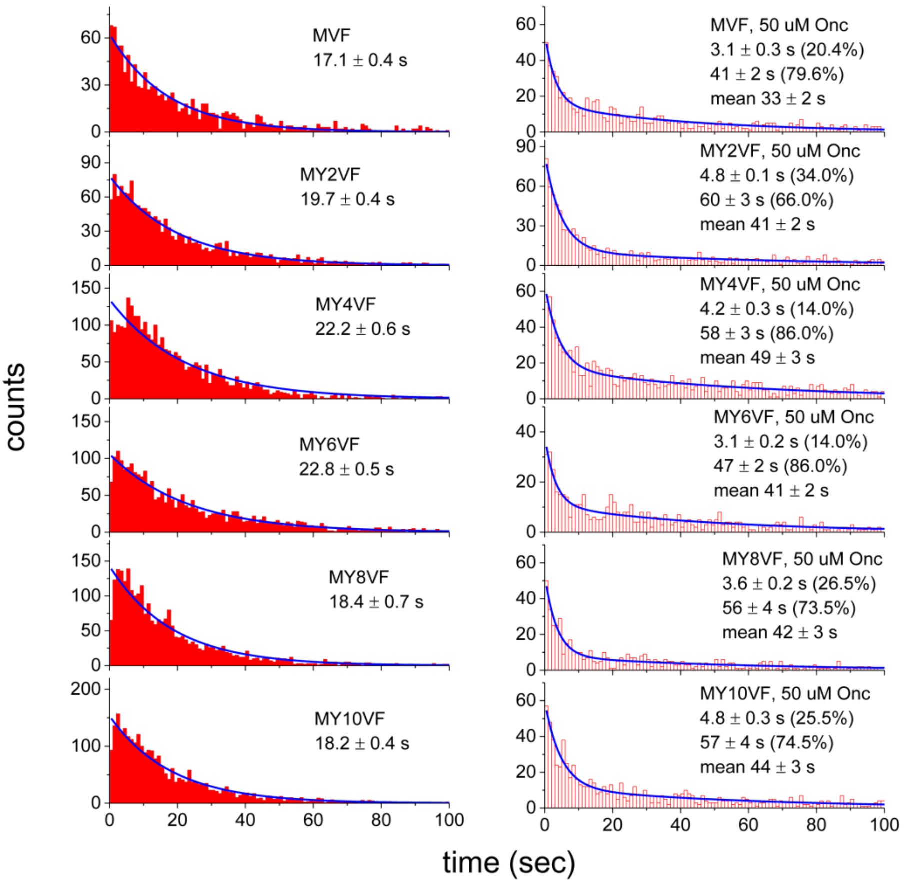

Fig. S1. Dwell time distributions of the Cy5-only state in real-time ribosome translation traces measured using Cy5-V and Cy3-F ternary complexes (examples shown in Figs. 1A and B). Ribosomes programmed with six different mRNAs (Table S1) were used and their smFRET traces were recorded without (solid bars) and with (hollow bars) Onc112. Time from steady Cy5 signals appearance until steady Cy3 and FRET signals appearance was recorded for individual ribosomal complexes and plotted as the dwell time distributions. Time constants were calculated using either single or double exponential decays. For double exponential decay, proportions of each component and weighted averaged were listed with S.E. of fitting results. Number of events included are 1160, 1551, 2863, 2416, 2554, and 2739 (from top to bottom, left column) and 791, 1023, 1167, 647, 637, and 907 (from top to bottom, right column).

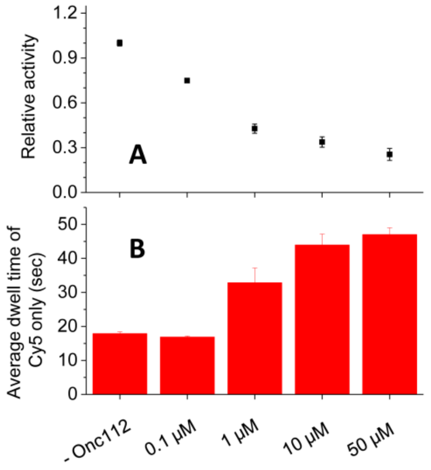

Fig. S2. tRNA-tRNA smFRET reveals how Onc112 inhibits elongation at different concentrations. The same smFRET assays as **Figs. 1A-1D** were used. (**A**) Relative activity of ribosomes translating to the VF motif of mRNA MY6VF. (**B**) Average dwell times of the Cy5-only state at different concentrations of Onc112 for mRNA MY6VF.

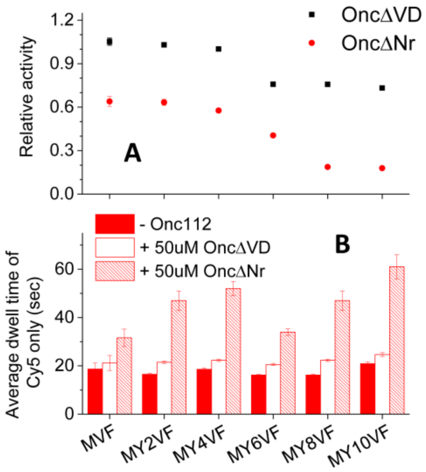

Fig. S3. tRNA-tRNA smFRET reveals how OncΔNr and OncΔVD inhibit elongation. The same smFRET assays as **Figs. 1A-1D** were used. (**A**) Relative activity of ribosomes translating on six different mRNAs to the VF motif. (**B**) Average dwell times of the Cy5-only state in the absence and presence of OncΔNr or OncΔVD for six different mRNAs.

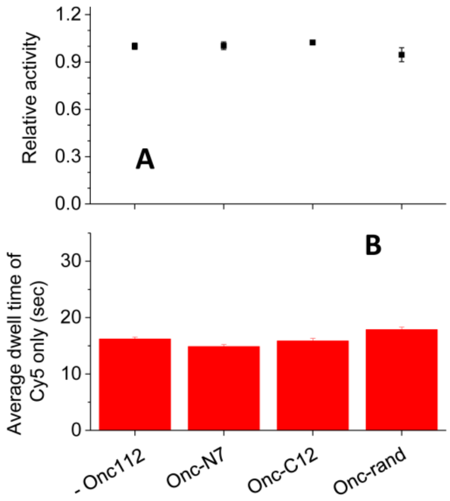

Fig. S4. tRNA-tRNA smFRET reveals how control peptides, Onc-N7, Onc-C12, and Onc-rand, affect elongation. The same smFRET assays as **Figs. 1A-1D** were used. (**A**) Relative activity of ribosomes translating to the VF motif of mRNA MY6VF. (**B**) Average dwell times of the Cy5-only state in the absence and presence of peptides for mRNA MY6VF.

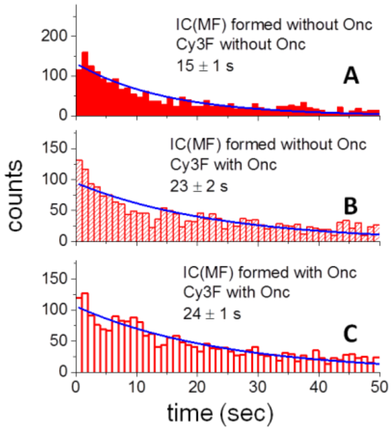

Fig. S5. Inhibition of aa-tRNA delivery by Onc112 in the first elongation cycle, characterized by dwell time distributions of initiation complexes (ICs) before Cy3-F accommodation. ICs, programmed with mRNA MF, containing Cy5-L11 were pre-formed in the absence (**A and B**) or presence (**C**) of Onc112. ICs were immobilized and incubated with Onc112 for three minutes (**B and C**). Then reaction mixtures containing 8 nM Cy3-F ternary complex without (**A**) or with (**B and C**) additional 50 μM Onc112 were injected onto ICs while collecting fluorescence signals. Time from injection until steady Cy3 and FRET signals appearance was recorded for individual ribosomal complexes and plotted as the dwell time distributions. Time constants calculated by exponential fitting of dwell time distributions were listed with S.E. of fitting results. Number of events included are 2066 (**A**), 2072 (**B**), and 2322 (**C**).

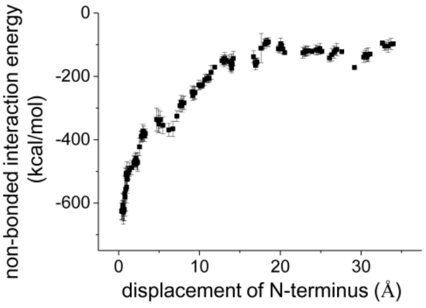

Fig. S6. Non-bonded interaction energy between Onc112 and peptide exit tunnel against the traveling distance of the N-terminus of Onc112 peptide (compared with crystal structure, which is defined as 0 displacement) towards exit site estimated by MD simulation.

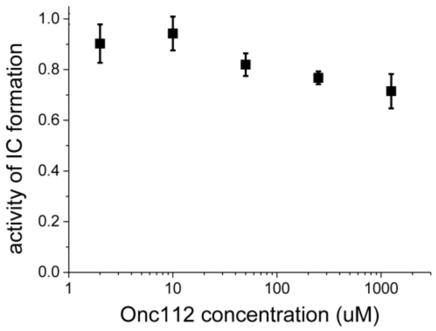

Fig. S7. Relative activity to form initiation complex (IC) in the presence of Onc112. ICs were formed by mixture of 70S-L11^{Cy5} ribosomes with biotinylated mRNA MVF, initiation factors, and tRNA^{fMet} in TAM$_{15}$ buffer with or without Onc112 at 37 ℃ for 25 min. Formation of ICs was characterized by incubating 1 nM IC with PEG-passivated streptavidin-decorated slides for 3 min, washing away unbound IC, and quantifying remaining Cy5 fluorescence spots on the slides. Under our experimental design, only 70S-L11^{Cy5} ribosomes formed into ICs with biotinylated mRNA can remain on the slides after washing; whereas free 70S-L11^{Cy5} ribosomes were washed away and had no contribution to number of Cy5 fluorescence spots. Number of Cy5 fluorescence spots in the absence of Onc112 was normalized to 1.

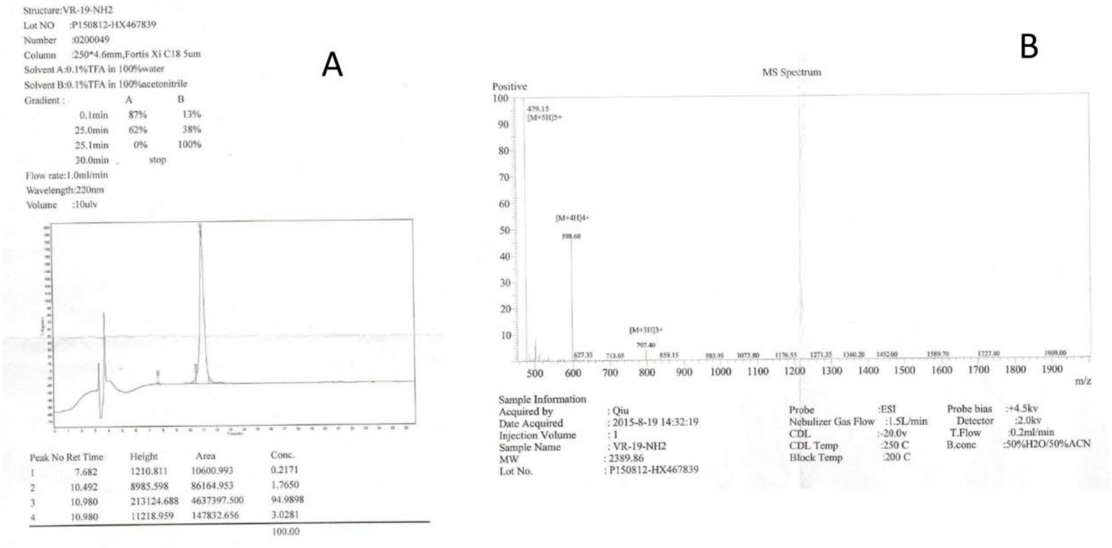

Fig. S8. HPLC (**A**) and mass spectrum (**B**) of Onc112 to verify its purity and quality.
